# Serum calcium and risk of gastrointestinal cancer in the Swedish AMORIS study

**DOI:** 10.1186/1471-2458-13-663

**Published:** 2013-07-17

**Authors:** Wahyu Wulaningsih, Karl Michaelsson, Hans Garmo, Niklas Hammar, Ingmar Jungner, Göran Walldius, Mats Lambe, Lars Holmberg, Mieke Van Hemelrijck

**Affiliations:** 1 King’s College London, School of Medicine, Division of Cancer Studies, Cancer Epidemiology Group, London, UK; 2 Department of Surgical Sciences, Uppsala University, Uppsala, Sweden; 3 Regional Cancer Centre, Uppsala University, Uppsala, Sweden; 4 Department of Epidemiology, Insitute of Environmental Medicine, Karolinska Institute, Stockholm, Sweden; 5 AstraZeneca Sverige, Södertalje, Sweden; 6 Department of Medicine, Clinical Epidemiological Unit, Karolinska Institutet and CALAB Research, Stockholm, Sweden; 7 Department of Medical Epidemiology and Biostatistics, Karolinska Institutet, Stockholm, Sweden; 8 King’s College London, School of Medicine, Division of Cancer Studies, Cancer Epidemiology Group, Research Oncology, 3rd Floor, Bermondsey Wing, Guy’s Hospital, London SE1 9RT, UK

**Keywords:** Gastrointestinal cancer, Calcium, Albumin

## Abstract

**Background:**

Observational studies have indicated that high calcium intake may prevent colorectal cancer, but as for randomized trials the results are inconclusive. Meanwhile, limited data on the link between serum calcium and cancer risk is available. We investigated the relation between serum calcium and risk of different gastrointestinal cancers in a prospective study.

**Methods:**

A cohort based on 492,044 subjects with baseline information on calcium (mmol/L) and albumin (g/L) was selected from the Swedish Apolipoprotein MOrtality RISk (AMORIS) study. Multivariable Cox proportional hazard models were used to analyse associations between standardised levels, quartiles and age/sex-specific categories of serum calcium and risk of oesophageal, stomach, colon, rectal cancer and also colorectal cancer combined, while taking into account serum albumin and other comorbidities.

**Results:**

During 12 years of follow-up, we identified 323 incident oesophageal cancers, 782 stomach cancers, 2519 colon cancers, and 1495 rectal cancers. A positive association was found between albumin-adjusted serum calcium and risk of oesophageal [HR: 4.82 (95% CI: 2.07 – 11.19) for high compared to normal age-specific calcium levels] and colon cancer [e.g. HR: 1.07 (95% CI: 1.00 – 1.14) for every SD increase of calcium] as well as colorectal cancer [e.g. HR: 1.06 (95% CI: 1.02-1.11) for every SD increase of calcium] in women. In men there were similar but weaker non-statistically significant trends.

**Conclusion:**

The positive relation between serum calcium, oesophageal cancer and colorectal cancer calls for further studies including calcium regulators to evaluate whether there is a true link between calcium metabolism and development of gastrointestinal cancer.

## Background

A role of dietary calcium in colorectal cancer prevention has been suggested [1], but there is lack of epidemiological evidence linking serum calcium and gastrointestinal cancer risk. High calcium intake has been shown to suppress cell cycle, promote apoptosis and reduce formation of colonic tumour in animal studies [2]. A pooled analysis including 534,536 individuals from ten cohort studies also revealed a lower colorectal cancer risk with higher calcium intake [3]. However, a randomized trial (n = 36,282) showed that daily supplementation of calcium in combination with vitamin D for seven years had no effect on colorectal cancer incidence among postmenopausal women [4]. Meanwhile, no clear association is known between dietary calcium intake and risk of oesophageal cancer. Since calcium homeostasis is mainly influenced by vitamin D and parathyroid hormone (PTH) instead of dietary calcium [5], the use of serum calcium may enable further insight into the relation between calcium metabolism and cancer risk. However, most available studies used uncorrected serum calcium, whilst calcium levels are influenced by serum albumin [6]. As calcium has also been linked to other diseases [7], to what extent serum calcium is related to the risk of gastrointestinal cancer while taking into account serum albumin and other co-morbidities warrants further investigation.

## Methods

### Study population and data collection

The Swedish AMORIS database has been described in detail elsewhere [8-14]. Briefly, this database is based on the linkage of the Central Automation Laboratory (CALAB) database (1985–1996) to several Swedish national registries such as the National Cancer Register, the National Patient Register, the Cause of Death Register, the consecutive Swedish Censuses during 1970–1990, and the National Register of Emigration by using the Swedish 10-digit personal identity number to provide information on socio-economic status (SES), vital status, cancer diagnosis, and emigration. The CALAB database includes data from 351,487 male and 338,101 female healthy individuals having clinical laboratory testing as part of a general health check-up or outpatients referred for laboratory testing. No individuals were inpatients at the time their blood samples were taken and none were excluded for disease symptoms or because of treatment. This study complied with the Declaration of Helsinki, and the ethics review board of the Karolinska Institute approved the study.

From the AMORIS database, we selected all persons aged 20 years or older at baseline, whose serum levels of calcium (mmol/L) and albumin (g/L) were measured at baseline (n = 492,044). Of these, a total of 63,028 had an additional baseline measurement of body-mass index (BMI, kg/m^2^). Follow-up time was defined for each subject as the time from measurement until the date of GI cancer diagnosis, emigration, death, or study closing date (31^st^ of December 2002), whichever occurred first. The CALAB database also contained information on age at index measurement. All other information was obtained from the above mentioned national registries. SES was taken from the Censuses and is based on occupational groups and classifies gainfully employed subjects into manual workers and non-manual employees, below designated blue-collar and white-collar workers [15]. We also calculated the Charlson co-morbidity index (CCI) by using the information from the National Patient Register. The CCI consists of 18 groups of diseases with a specific weight assigned to each disease category [1-3,6]. These weights were then summed to obtain an overall score, resulting in four co-morbidity levels (0, 1, 2, and 3+) indicating a scale ranging from no co-morbidity to severe co-morbidity.

Ionized serum calcium level is a direct measure of the amount of metabolically active serum calcium but is not routinely measured [16]. Correction of total calcium levels based on serum albumin is used to obtain an estimate of the free ionized calcium level, since almost half (40%) of serum calcium is in protein-bound form and alteration of serum albumin due to certain diseases or dietary patterns may thus affect the levels of free ionized calcium [5,16]. A colorimetric method was used for the measurement of total serum calcium (coefficient of variation <2.5%), whereas albumin was measured with a bromcresolgreen (BCG) method (coefficient of variation <1.8%). We corrected the calcium levels to the concentration as if the albumin level was normal, meaning that for every 1 g/L that the albumin concentration was below 40 g/L (normal concentration), the calcium concentration was increased with 0.02 mmol/L [16]. All levels were standardized according to the World Health Organization International Federation of Clinical Chemists protocols and all methods were fully automated with automatic calibration and performed at one accredited laboratory [8,17].

Levels of uncorrected calcium were also categorized into three categories (low, normal, high) based on whether it was below, equal, or above the reference interval used in CALAB laboratory. This reference interval differed by age and sex (Table 1).

**Table 1:** Reference levels of uncorrected calcium (mmol/L) by age and sex

	**Men**	**Women**
< 40 years	2.22-2.60	2.17-2.56
40-60 years	2.20-2.59	2.19-2.60
≥ 60 years	2.19-2.58	2.21-2.60

### Data analysis

The associations between levels of serum calcium, age-specific calcium, albumin-corrected calcium and risk of incident GI cancer were analyzed with multivariate Cox proportional hazards models. A test for trend was conducted by using assignment to categories as an ordinal scale. All models took into account age at index measurements. When studying the association between crude calcium and gastrointestinal cancer risk, we also adjusted the Cox proportional hazards models for albumin levels as a continuous variable. Additional adjustments were performed for gender, SES, and CCI. Analyses were repeated in men and women separately to further evaluate gender effects. We considered the probability of reverse causation (i.e. calcium levels can be affected by an undiagnosed gastrointestinal cancer) by conducting a sensitivity analysis in which the first three years of follow-up were excluded (n = 9,171). Additionally, we did a stratified analysis by BMI (</≥25 kg/m^2^), as it was shown previously that the association between calcium and cancer risk is possibly modified by levels of BMI [18].

Gastrointestinal cancer is a disease with a long natural history and mainly seen in the elderly, thus competing risks are involved in the analysis of gastrointestinal cancer risk. A competing risk situation arises when an individual can not only experience the event of interest, but also be censored due to other events [19]. It is even more important to consider competing risk when, as in this study, the exposure of interest may be linked to both gastrointestinal cancer and early death. Thus, death from other causes is considered to be competing risks for gastrointestinal cancer diagnosis. In a Cox proportional hazard model, as described above, one of the main assumptions is that censoring is non-informative. We violate this assumption when we censor for death (and not having gastrointestinal cancer) if death is related to our exposure of interest, calcium levels. To evaluate whether competing risks may be affecting our findings, we calculated proportional hazards models in which we censored for gastrointestinal cancer diagnosis and considered death as the outcome of interest. Moreover, we conducted a case-only survival analysis for gastrointestinal cancer outcomes, while also adjusting for the time between measurement of calcium and gastrointestinal cancer diagnosis. For these analyses, follow-up was defined as the time from gastrointestinal cancer diagnosis until the date of gastrointestinal cancer death, emigration, death, or study closing date (31 December 2002), whichever occurred first.

All P-values were two-sided and all analyses were conducted with Statistical Analysis Systems (SAS) release 9.1.3 (SAS Institute, Cary, NC).

## Results

A total of 323 cases of incident oesophageal cancers, 782 stomach cancers, 2519 colon cancers, 1495 rectal cancers, and a combined 4014 colorectal cancer were identified during follow-up (mean: 12.5 years). Study population characteristics are shown in Table 2. The Pearson correlation coefficient was 0.42 (P-value < 0.0001) for calcium and albumin and 0.81 (P-value < 0.0001) for uncorrected and albumin-corrected calcium.

**Table 2:** Description of study population by gastrointestinal cancer status

	**Oesophageal cancer**	**Stomach cancer**	**Colon cancer**	**Rectal cancer**	**Colorectal cancer**	**All**
	***(n = 323)***	***(n = 782)***	***(n = 2519)***	***(n = 1495)***	***(n = 4014)***	***(n = 492044)***
**Age (years) -** Mean (SD)	56.07 (10.63)	58.72 (11.80)	58.93 (11.61)	57.38 (11.02)	58.36 (11.42)	44.62 (14.11)
**Sex**						
Male	237 (71.39)	501 (62.47)	1394 (54.09)	926 (60.76)	2320 (56.57)	260469 (52.94)
Female	95 (28.61)	301 (37.53)	1183 (45.91)	598 (39.24)	1781 (43.43)	231575 (47.06)
**SES**						
White Collar	112 (33.73)	262 (32.67)	946 (36.71)	583 (38.25)	1529 (37.28)	182488 (37.09)
Blue Collar	162 (48.80)	359 (44.76)	1063 (41.25)	684 (44.88)	1747 (42.60)	231088 (46.96)
Not gainfully employed or Missing	58 (17.47)	181 (22.57)	568 (22.04)	257 (16.86)	825 (20.12)	78468 (15.95)
**Follow-up time (years) -** Mean (SD)	8.06 (4.71)	7.73 (4.62)	8.46 (4.64)	8.44 (4.51)	8.46 (4.59)	12.55 (3.94)
**Calcium (mmol/L) -** Mean (SD)	2.40 (0.11)	2.38 (0.10)	2.39 (0.10)	2.39 (0.10)	2.39 (0.10)	2.39 (0.10)
**Calcium according to age-specific cut-offs**						
Low	2 (0.60)	9 (1.12)	17 (0.66)	13 (0.85)	30 (0.73)	4025 (0.82)
Normal	320 (96.39)	778 (97.01)	2508 (97.32)	1477 (96.92)	3985 (97.17)	479026 (97.35)
High	10 (3.10)	15 (1.87)	52 (2.02)	34 (2.23)	86 (2.10)	8993 (1.83)
**Corrected Calcium (mmol/L) -** Mean (SD)	2.35 (0.10)	2.34 (0.09)	2.35 (0.09)	2.34 (0.09)	2.35 (0.09)	2.32 (0.09)
**Albumin (g/L)** Mean (SD)	42.11 (2.97)	41.82 (2.75)	41.92 (2.69)	42.28 (2.68)	42.05 (2.69)	43.18 (2.84)
**BMI (kg/m**^**2**^**)**^**1**^						
< 25	17 (58.62)	40 (50.63)	141 (50.72)	96 (55.49)	237 (52.55)	38517 (61.11)
≥ 25	12 (41.38)	39 (49.37)	137 (49.28)	77 (44.51)	214 (47.45)	24511 (38.89)
**Charlson Comorbidity Index**						
0	273 (82.23)	666 (83.04)	2199(85.33)	1312 (86.09)	3511 (85.61)	454237 (92.32)
1	30 (9.04)	77 (9.60)	190 (7.37)	113 (7.41)	303 (7.39)	20669 (4.20)
2	19 (5.72)	42 (5.24)	143 (5.55)	77 (5.05)	220 (5.36)	12869 (2.62)
3+	10 (3.01)	17 (2.12)	45 (1.75)	22 (1.44)	67 (1.63)	4269 (0.87)

^1^Measured in subcohort with BMI measurement.

First, to investigate the relation between calcium and gastrointestinal cancer, we used different measurements of calcium in age-adjusted Cox proportional hazard models (Table 3). An inverse trend was observed between quartiles of serum calcium and risk of stomach and colon cancers in the age-adjusted models [e.g. HR for colon cancer 0.92 (95% CI: 0.82 – 1.02), 0.94 (0.84 – 1.05), and 0.88 (0.79 – 0.98) for the 2^nd^, 3^rd^, and 4^th^ quartiles of calcium, respectively; P-value for trend 0.03]. A similar but borderline association was also seen between calcium and colorectal cancer, while no apparent link was observed for oesophageal cancer and rectal cancer alone. Interestingly, this inverse trend was not evident after adjustment for gender, SES, albumin, and CCI. For calcium based on age-specific cut-offs, a statistically significant association was only observed with colorectal cancer risk after adjustment with gender, SES, albumin, and CCI [HR: 1.27 (95% CI: 1.03-1.58) for those with high age-specific calcium levels compared to normal].

**Table 3:** Hazard ratios and 95% confidence intervals (CI) for the association between calcium (age-specific and albumin-corrected) and risk of gastrointestinal cancer

	**Oesophageal cancer**			**Stomach cancer**			**Colon cancer**			**Rectal cancer**			**Colorectal cancer**		
	**n**	**HR (95% CI)**	**HR (95% CI)**^**1**^	**n**	**HR (95% CI)**	**HR (95% CI)**^**1**^	**n**	**HR (95% CI)**	**HR (95% CI)**^**1**^	**n**	**HR (95% CI)**	**HR (95% CI)**^**1**^	**n**	**HR (95% CI)**	**HR (95% CI)**^**1**^
**Calcium** (SD: 0.10 mmol/L)		1.07 (0.96-1.18)	1.16 (1.04-1.30)		0.91 (0.85-0.97)	0.98 (0.91-1.06)		0.97 (0.94-1.01)	1.04 (0.99-1.08)		1.00 (0.95-1.05)	1.03 (0.97-1.09)		0.98 (0.95-1.01)	1.04 (1.01-1.07)
**Quartiles of calcium**															
< 2.32	71	1.00 (Ref)	1.00 (Ref)	187	1.00 (Ref)	1.00 (Ref)	575	1.00 (Ref)	1.00 (Ref)	314	1.00 (Ref)	1.00 (Ref)	989	1.00 (Ref)	1.00 (Ref)
2.32-2.38	76	0.91 (0.67-1.24)	0.97 (0.71-1.32)	218	0.96 (0.80-1.16)	1.03 (0.85-1.25)	622	0.92 (0.82-1.02)	0.96 (0.86-1.07)	379	1.13 (0.98-1.31)	1.14 (0.99-1.32)	1001	0.99 (0.91-1.08)	1.03 (0.94-1.12)
2.38-2.45	76	0.86 (0.63-1.19)	0.96 (0.69-1.34)	171	0.78 (0.64-0.96)	0.88 (0.72-1.09)	665	0.94 (0.84-1.05)	1.02 (0.91-1.15)	415	1.10 (0.95-1.27)	1.12 (0.97-1.31)	1080	0.99 (0.91-1.08)	1.06 (0.97-1.16)
≥ 2.45	100	1.08 (0.81-1.44)	1.31 (0.96-1.80)	206	0.86 (0.72-1.04)	1.06 (0.86-1.29)	657	0.88 (0.79-0.98)	1.01 (0.90-1.14)	387	0.99 (0.86-1.13)	1.04 (0.89-1.21)	1044	0.92 (0.84-1.00)	1.02 (0.93-1.12)
P-value for trend		0.61	0.09		0.04	0.95		0.03	0.56		0.08	0.71		0.05	0.51
**Calcium according to age-specific cut-offs**															
Low	2	0.77 (0.19-2.08)	0.64 (0.16-2.58)	9	1.34 (0.70-2.59)	1.09 (0.56-2.12)	17	0.81 (0.50-1.31)	0.69 (0.43-1.12)	13	1.08 (0.62-1.86)	1.06 (0.61-1.84)	30	0.91 (0.63-1.30)	0.82 (0.57-1.17)
Normal	311	1.00 (Ref)	1.00 (Ref)	758	1.00 (Ref)	1.00 (Ref)	2453	1.00 (Ref)	1.00 (Ref)	1449	1.00 (Ref)	1.00 (Ref)	3902	1.00 (Ref)	1.00 (Ref)
High	10	1.73 (0.92-3.24)	1.95 (1.03-3.69)	15	1.04 (0.62-1.73)	1.19 (0.71-1.99)	49	1.12 (0.85-1.48)	1.25 (0.95-1.65)	33	1.27 (0.90-1.78)	1.30 (0.92-1.84)	82	1.18 (0.95-1.46)	1.27 (1.03-1.58)
**Albumin-corrected calcium**^**2**^															
(SD: 0.09 mmol/L)		1.14 (1.03-1.26)	1.17 (1.05-1.30)		0.98 (0.92-1.05)	1.00 (0.94-1.08)		1.03 (0.99-1.07)	1.05 (1.01-1.09)		1.01 (0.96-1.06)	1.03 (0.98-1.08)		1.02 (0.99-1.05)	1.04 (1.01-1.07)
**Quartiles of albumin-corrected calcium**^**2**^															
< 2.27	59	1.00 (Ref)	1.00 (Ref)	149	1.00 (Ref)	1.00 (Ref)	404	1.00 (Ref)	1.00 (Ref)	272	1.00 (Ref)	1.00 (Ref)	676	1.00 (Ref)	1.00 (Ref)
2.27-2.33	76	0.88 (0.61-1.25)	0.86 (0.61-1.25)	198	0.96 (0.77-1.19)	0.96 (0.77-1.20)	640	1.12 (0.99-1.27)	1.13 (0.99-1.28)	385	1.03 (0.88-1.21)	1.04 (0.88-1.22)	1025	1.09 (0.98-1.20)	1.09 (0.99-1.21)
2.33-2.39	78	1.03 (0.74-1.44)	1.04 (0.75-1.44)	186	0.89 (0.72-1.11)	0.91 (0.73-1.12)	668	1.09 (0.97-1.24)	1.10 (0.98-1.25)	398	1.05 (0.90-1.21)	1.06 (0.91-1.24)	1066	1.08 (0.98-1.19)	1.09 (0.99-1.20)
≥ 2.39	110	1.20 (0.88-1.63)	1.26 (0.92-1.72)	249	0.97 (0.79-1.18)	1.02 (0.83-1.24)	807	1.16 (1.03-1.30)	1.19 (1.06-1.34)	440	0.99 (0.86-1.15)	1.04 (0.90-1.21)	1247	1.09 (1.00-1.20)	1.13 (1.03-1.24)
P-value for trend		0.11	0.05		0.72	0.86		0.03	0.007		0.99	0.61		0.12	0.02

All models are adjusted for age at index measurement.

^1^Adjusted for sex, SES, albumin (continuous), and Charlson comorbidity index.

^2^Not adjusted for albumin.

When using albumin-corrected calcium, we found a higher risk of colon cancer in those in higher albumin-corrected calcium quartiles., Additional adjustments for gender, SES, and CCI resulted in a stronger association with colon cancer risk [HR for colon cancer: 1.13 (95% CI: 0.99 – 1.28), 1.10 (0.98 – 1.28), and 1.16 (1.06 – 1.34) for the 2^nd^, 3^rd^, and 4^th^ quartiles of calcium, respectively; P-value for trend 0.007] and a statistically significant association with colorectal cancer risk [e.g. HR for colorectal cancer: 1.09 (95% CI: 0.99 – 1.21), 1.09 (0.99 – 1.20), and 1.13 (1.03 – 1.34) for the 2^nd^, 3^rd^, and 4^th^ quartiles of calcium, respectively; P-value for trend 0.02].

When analyzing men and women separately while adjusting for serum albumin, we observed a similar but non-statistically significant relation with serum calcium for men (Table 4). For women, those with high age-specific calcium levels are at higher risks for developing oesophageal cancer [e.g. HR: 4.82 (95% CI: 2.07-11.19) for high age-specific calcium compared to normal]. Similar to the results in men and women combined, a statistically significant association with risk of colon cancer was observed in women [HR: 1.07 (95% CI: 1.00 – 1.14) for every SD increase in serum calcium]. Using albumin-corrected calcium, we found a positive link with with colon as well as colorectal cancer risk in women [e.g. HR for colon and colorectal cancer: 1.07 (95% CI: 1.02-1.13) and 1.06 (95% CI: 1.02-1.11) for every SD increase in corrected calcium, respectively]. A sensitivity analysis excluding those with follow-up less than 3 years did not substantially alter these findings (results not shown).

**Table 4:** Hazard ratios and 95% confidence intervals (CI) for the association between calcium and risk of gastrointestinal cancer in men and women

**Men *(N = 260,469)***										
	**Oesophageal cancer**		**Stomach cancer**		**Colon cancer**		**Rectal cancer**		**Colorectal cancer**	
	**n**	**HR (95% CI)**	**n**	**HR (95% CI)**	**n**	**HR (95% CI)**	**n**	**HR (95% CI)**	**n**	**HR (95% CI)**
**Calcium** (SD: 0.10 mmol/L)		1.13 (0.98-1.30)		0.94 (0.85-1.04)		1.02 (0.96-1.08)		1.03 (0.96-1.11)		1.02 (0.98-1.07)
**Quartiles of calcium**										
< 2.33	62	1.00 (Ref)	143	1.00 (Ref)	389	1.00 (Ref)	223	1.00 (Ref)	612	1.00 (Ref)
2.33-2.39	62	0.89 (0.62-1.27)	171	1.13 (0.90-1.41)	407	0.96 (0.84-1.11)	282	1.10 (0.92-1.32)	689	1.01 (0.91-1.13)
2.39-2.45	57	1.00 (0.68-1.45)	99	0.84 (0.64-1.09)	352	1.05 (0.90-1.22)	236	1.12 (0.92-1.35)	588	1.07 (0.96-1.21)
≥ 2.45	56	1.25 (0.84-1.86)	88	0.99 (0.74-1.31)	246	0.96 (0.81-1.15)	185	1.10 (0.89-1.36)	431	1.02 (0.89-1.16)
P-value for trend		0.24		0.40		0.97		0.36		0.55
**Calcium according to age-specific cut-offs**										
Low	1	0.56 (0.08-4.00)	4	0.92 (0.34-2.48)	7	0.62 (0.29-1.30)	9	1.47 (0.78-2.86)	16	1.01 (0.97-1.06)
Normal	232	1.00 (Ref)	487	1.00 (Ref)	1360	1.00 (Ref)	895	1.00 (Ref)	2255	1.00 (Ref)
High	4	1.03 (0.38-2.78)	10	1.27 (0.67-2.38)	27	1.21 (0.83-1.78)	22	1.36 (0.89-2.09)	49	0.92 (0.56-1.51)
**Albumin-corrected calcium**^**1**^										
(SD: 0.09 mmol/L)		1.14 (1.00-1.29)		0.98 (0.89-1.07)		1.04 (0.98-1.10)		1.03 (0.97-1.11)		1.04 (0.99-1.08)
**Quartiles of albumin-corrected calcium**^**1**^										
< 2.27	50	1.00 (Ref)	110	1.00 (Ref)	258	1.00 (Ref)	187	1.00 (Ref)	445	1.00 (Ref)
2.27-2.33	40	0.75 (0.49-1.13)	106	0.89 (0.68-1.16)	316	1.12 (0.95-1.32)	204	1.01 (0.83-1.23)	520	1.08 (0.95-1.22)
2.33-2.38	64	0.97 (0.67-1.40)	134	0.91 (0.71-1.17)	378	1.09 (0.93-1.28)	260	1.05 (0.87-1.26)	638	1.07 (0.95-1.21)
≥ 2.38	83	1.16 (0.82-1.65)	151	0.93 (0.73-1.19)	442	1.17 (1.00-1.36)	275	1.02 (0.85-1.23)	727	1.11 (0.98-1.25)
P-value for trend		0.17		0.68		0.08		0.76		0.12
**Women *(N = 231,575)***										
**Calcium** (SD: 0.10 mmol/L)		1.25 (1.04-1.51)		1.07 (0.95-1.20)		1.07 (1.00-1.14)		1.04 (0.96-1.13)		1.06 (1.01-1.11)
**Quartiles of calcium**										
< 2.32	21	1.00 (Ref)	58	1.00 (Ref)	211	1.00 (Ref)	113	1.00 (Ref)	324	1.00 (Ref)
2.32-2.38	23	0.98 (0.54-1.79)	77	1.12 (0.79-1.58)	302	1.17 (0.98-1.40)	147	1.04 (0.82-1.34)	449	1.13 (0.98-1.30)
2.38-2.44	18	0.80 (0.42-1.55)	66	0.98 (0.68-1.41)	300	1.17 (0.97-1.40)	160	1.12 (0.88-1.44)	460	1.15 (0.99-1.33)
≥ 2.44	33	1.30 (0.71-2.38)	100	1.25 (0.88-1.77)	370	1.18 (0.99-1.42)	178	1.01 (0.78-1.30)	548	1.12 (0.97-1.30)
P-value for trend		0.32		0.30		0.13		0.90		0.19
**Calcium according to age-specific cut-offs**										
Low	1	0.70 (0.10-5.16)	5	1.44 (0.59-3.53)	10	0.76 (0.41-1.43)	4	0.66 (0.25-1.78)	14	0.73 (0.43-1.24)
Normal	88	1.00 (Ref)	291	1.00 (Ref)	1148	1.00 (Ref)	582	1.00 (Ref)	1730	1.00 (Ref)
High	6	4.82 (2.07-11.19)	5	1.02 (0.42-2.48)	25	1.30 (0.87-1.94)	12	1.19 (0.67-2.11)	37	1.26 (0.91-1.75)
**Albumin-corrected calcium**^**1**^										
(SD: 0.09 mmol/L)		1.26 (1.06-1.50)		1.08 (0.97-1.20)		1.07 (1.02-1.13)		1.04 (0.96-1.12)		1.06 (1.02-1.11)
**Quartiles of albumin-corrected calcium**^**1**^										
< 2.27	9	1.00 (Ref)	36	1.00 (Ref)	134	1.00 (Ref)	75	1.00 (Ref)	209	1.00 (Ref)
2.27-2.32	20	1.46 (0.67-3.22)	67	1.20 (0.80-1.80)	250	1.17 (0.95-1.45)	133	1.15 (0.86-1.52)	383	1.16 (0.98-1.38)
2.32-2.38	23	1.45 (0.66-3.15)	66	1.00 (0.66-1.51)	302	1.19 (0.97-1.47)	159	1.18 (0.89-1.55)	461	1.19 (1.01-1.40)
≥ 2.38	43	1.87 (0.89-3.91)	132	1.34 (0.92-1.96)	497	1.32 (1.08-1.60)	231	1.17 (0.89-1.53)	728	1.26 (1.08-1.48)
P-value for trend		0.09		0.15		0.007		0.34		0.006

All models are adjusted for age, SES, albumin (continuous), and Charlson comorbidity index.

^1^Not adjusted for albumin.

Next, we assessed the risk of death as a competing risk while censoring for gastrointestinal cancer in the models. We found a statistically significant association between serum calcium and risk of death [e.g. HR: 1.14 (1.13 – 1.15) for every SD increase in calcium; results not shown in tables]. When using the age-specific calcium levels, we observed higher risks of death in those with both low and high serum calcium [HR: 1.26 (95% CI: 1.15 – 1.37) and 1.47 (1.39 – 1.57) for low and high age-specific calcium levels, respectively].

We also investigated the relation between calcium and gastrointestinal cancer risk while stratifying for overweight status (</≥25 kg/m^2^) in a subcohort with baseline BMI. However this resulted in low statistical power of our analyses (Table 5). A statistically significant association was only observed for oesophageal cancer in those with BMI < 25 kg/m^2^ [HR: 1.92 (95% CI: 1.32-2.82) for every SD increase in albumin-corrected calcium].

**Table 5:** **Hazard ratios and 95% confidence intervals (CI) for the association between calcium and risk of GI cancer, stratified by overweight (BMI</≥25 kg/m**^**2**^**) in sub-cohort with baseline measurements of BMI**

**Not Overweight *(N = 24,511)***										
	**Oesophageal cancer**		**Stomach cancer**		**Colon cancer**		**Rectal cancer**		**Colorectal cancer**	
	**n**	**HR (95% CI)**	**n**	**HR (95% CI)**	**n**	**HR (95% CI)**	**n**	**HR (95% CI)**	**n**	**HR (95% CI)**
**Calcium** (SD: 0.10 mmol/L)		2.02 (1.33-3.07)		0.81 (0.57-1.14)		1.01 (0.84-1.21)		1.10 (0.88-1.38)		1.04 (0.91-1.21)
**Quartiles of calcium**										
< 2.32	3	1.00 (Ref)	13	1.00 (Ref)	34	1.00 (Ref)	18	1.00 (Ref)	52	1.00 (Ref)
2.32-2.38	3	1.04 (0.20-5.30)	11	0.67 (0.28-1.51)	42	1.08 (0.68-1.71)	28	1.72 (0.72-2.38)	70	1.05 (0.91-1.21)
2.38-2.45	4	1.86 (0.39-8.87)	7	0.53 (0.20-1.37)	33	1.07 (0.65-1.75)	30	1.74 (0.95-3.20	63	1.15 (0.87-1.52)
≥ 2.45	7	3.28 (0.73-14.71)	9	0.58 (0.23-1.47)	32	0.91 (0.54-1.55)	20	1.03 (0.52-2.04)	52	0.57 (0.34-0.96)
P-value for trend		0.07		0.21		0.73		0.74		0.95
**Albumin-corrected calcium**^**1**^		1.93 (1.32-2.82)		1.03 (0.61-1.14)		1.02 (0.86-1.21)		1.10 (0.90-1.35)		1.05 (0.93-1.20)
(SD: 0.09 mmol/L)										
**Quartiles of albumin-corrected calcium**^**1**^										
< 2.27	2	1.00 (Ref)	8	1.00 (Ref)	23	1.00 (Ref)	16	1.00 (Ref)	39	1.00 (Ref)
2.27-2.33	2	0.90 (0.13-6.84)	10	1.08 (0.42-2.73)	31	1.20 (0.70-2.05)	19	1.03 (0.53-2.00)	50	1.13 (0.74-1.71)
2.33-2.39	3	1.06 (0.18-6.37)	12	1.04 (0.42-2.55)	42	1.30 (0.78-2.16)	29	1.26 (0.68-2.33)	71	1.28 (0.87-1.90)
≥ 2.39	10	2.94 (0.64-13.65)	10	0.71 (0.28-1.80)	45	1.12 (0.67-1.86)	32	1.14 (0.62-2.09)	87	1.13 (0.76-1.66)
P-value for trend		0.07		0.42		0.72		0.58		0.53
Overweight ***(N = 38,517)***										
**Calcium** (SD: 0.10 mmol/L)		0.69 (0.38-1.28)		1.04 (0.74-1.46)		0.97 (0.81-1.16)		0.98 (0.77-1.25)		0.97 (0.84-1.13)
**Quartiles of calcium**										
< 2.31	5	1.00 (Ref)	11	1.00 (Ref)	35	1.00 (Ref)	18	1.00 (Ref)	53	1.00 (Ref)
2.31-2.37	3	0.61 (0.14-2.61)	8	0.66 (0.26-1.65)	35	0.85 (0.53-1.37)	22	1.01 (0.54-1.90)	57	0.91 (0.60-1.32)
2.37-2.44	1	0.27 (0.03-2.36)	11	1.08 (0.45-2.58)	39	1.09 (0.68-1.75)	16	0.82 (0.41-1.64)	55	0.99 (0.67-1.47)
≥ 2.44	3	1.00 (0.21-4.78)	9	1.00 (0.38-2.60)	28	0.83 (0.48-1.42)	21	1.09 (0.55-2.17)	49	0.92 (0.61-1.41)
P-value for trend		0.72		0.52		0.76		0.94		0.85
**Albumin-corrected calcium**^**1**^										
(SD: 0.09 mmol/L)		0.77 (0.44-1.35)		1.06 (0.78-1.45)		0.99 (0.84-1.17)		0.99 (0.79-1.23)		0.99 (0.86-1.13)
**Quartiles of albumin-corrected calcium**^**1**^										
< 2.27	3	1.00 (Ref)	8	1.00 (Ref)	24	1.00 (Ref)	15	1.00 (Ref)	39	1.00 (Ref)
2.27-2.33	3	0.77 (0.15-3.82)	7	0.65 (0.24-1.79)	31	0.94 (0.55-1.60)	15	0.72 (0.35-1.48)	46	0.85 (0.56-1.31)
2.33-2.39	3	0.73 (0.15-3.65)	9	0.81 (0.31-2.10)	43	1.26 (0.76-2.08)	23	1.08 (0.56-2.08)	66	1/13 (0.80-1.77)
≥ 2.39	3	0.65 (0.13-3.29)	15	1.15 (0.48-2.75)	39	0.95 (0.57-1.59)	24	0.92 (0.48-1.77)	63	0.94 (0.63-1.41)
P-value for trend		0.62		0.52		0.90		0.84		0.83

All models are adjusted for age, sex, SES, albumin (continuous), and Charlson Comorbidity Index.

^1^ Not adjusted for albumin.

Finally, we estimated risk of fatal gastrointestinal cancer by assessing risk of cancer-specific death in those diagnosed with gastrointestinal cancer. No statistically significant association was found in these models (results not shown). We also performed a sensitivity analysis for gastrointestinal cancer diagnosed < 3 years after baseline measurements, but this did not affect our results (results not shown).

## Discussion

The present study showed a lower risk of colon cancer in those in higher serum calcium quartiles but higher risk for rectal cancer in those with high age-specific calcium levels. However, correction with albumin or adjustment for gender, SES, albumin and CCI revealed a positive link between serum calcium and colon as well as colorectal cancer risk, particularly in women. Additionally, we found a persistent positive link between serum calcium and oesophageal cancer risk, but this trend was not observed in overweight individuals.

The role of diet in the aetiology of gastrointestinal malignancies has been an emerging subject of research. In animal studies, a Western-style diet with decreased calcium and vitamin D was shown to induce formation of intestinal tumours in normal mice during long-term observation, although no marked difference was seen from those treated with a standard diet [20,21]. Low calcium diets have been linked to enhanced cyclin D1 and Bcl-2 expression and decreased Bax expression, suggesting the modulation of cell cycle and apoptosis to play a part in increased tumourigenesis [2]. Dietary calcium may affect gastrointestinal malignancy directly via activation of calcium-sensing receptor (CaR) and binding of bile acids in gastrointestinal tract, and indirectly by increasing serum calcium levels [1,22]. A dual effect of luminal calcium on colon cells have been observed, where it promotes differentiation and apoptosis of normal colon cells progenitor, but becomes ineffective or even tumor-promoting in carcinogenesis [23]. On the other hand, how exactly serum calcium influences carcinogenesis remains indefinite. It is suggested that the rise of extracellular calcium results in increased cytosolic levels of calcium, which subsequently affects multiple cellular processes including cell cycle and apoptosis, possibly via Ras and β-catenin pathways [22,24].

Despite the above pre-clinical findings, the effects of calcium metabolism on gastrointestinal cancer development in human are not fully understood yet. Extensive epidemiological evidence has indicated the protective role of dietary calcium against both colorectal adenoma and invasive colorectal cancer [3,25-27]. However little evidence exists to explain the effects of serum calcium on gastrointestinal cancer, or whether similar effects on cancer risks are shared by dietary and serum calcium. In clinical studies, calcium supplementation in persons with resected colorectal adenomatous polyp has been linked to increased Bax expression in polyp interiors, suggesting enhanced apoptosis as the mechanism opposing carcinogenesis [28]. Randomized controlled trials of colorectal adenoma patients treated with calcium and vitamin D supplementation showed similar findings [5,29], as well as a lower colorectal adenoma recurrence compared to the placebo group [23]. On the other hand, a meta-analysis assessing two clinical trials with supplementation of calcium and vitamin D in population without increased baseline risks revealed no difference in colorectal cancer incidence between post-menopausal women with and without treatment [30], indicating different mechanisms linking calcium to colorectal adenoma and invasive cancer albeit not confirmed using serum calcium yet.

Calcium was statistically significantly correlated to albumin in the present study, which may explain the different patterns of association with and without adjustment or correction of serum calcium. After taking albumin into account, a positive association was observed between serum calcium and risk of oesophageal and colon cancer in women, as opposed to the suggested inverse relation between dietary calcium and colorectal cancer, and a lack of association with oesophageal cancer [3,31]. Although dietary calcium is a major determinant of total body calcium balance, serum calcium reflects extracellular calcium homeostasis and is mainly affected by vitamin D and PTH, thus abnormalities in serum calcium may reflect a defect in its regulation processes rather than lack of dietary calcium [5]. This may results in different associations between dietary and serum calcium and carcinogenesis. High serum levels of calcium may follow from hyperparathyroidism or high serum vitamin D levels which may then suppress production of another hormonal regulator, FGF23, and lead to down-regulation of klotho, a cancer prevention protein [32]. Furthermore, a true inverse association may have been masked as high serum calcium is associated with death from other diseases such as cardiovascular diseases [7].

In the present study, we assessed risk of death while censoring those diagnosed with gastrointestinal cancer. We found a strong association between calcium and risk of death from other diseases, indicating that gastrointestinal cancer is competing with other causes of death. This highlighted the importance of including other comorbidities as measured by CCI in the analyses to minimize the effects of competing risk on the association between calcium and gastrointestinal cancer.

Corroborating the suggested link between calcium and obesity in the context of cancer [33], we found markedly different risk estimates for oesophageal cancer when stratifying according to overweight status [e.g. HR: 1.93 (95% CI: 1.32-2.82) and 0.77 (0.44-1.35) for every SD increase of albumin-corrected calcium in normoweight and overweight individuals, respectively]. However the limited samples resulted in a low statistical power in this analysis, which explained weaker associations observed in this subcohort compared to the overall study population. Therefore increasing the number of samples with available baseline BMI is essential in studying the link between calcium, obesity, and gastrointestinal cancer in future studies.

We also investigated risk of fatal gastrointestinal cancer using cancer-specific death as an outcome in those diagnosed with gastrointestinal cancer. No apparent link between different measurements of calcium and gastrointestinal cancer death was observed, which may be partly a result of small number of fatal cancer in these subgroups. Additionally, there is a considerable lag time between baseline measurements and diagnosis of gastrointestinal cancer. Calcium measurements closer to diagnosis date may provide a better assessment of gastrointestinal cancer death, but also at risk of being influenced by the course of the disease.

The major strength of these analyses lies in the large number of men with prospective measurements of calcium, albumin, and glucose in AMORIS, all measured at the same clinical laboratory. To our knowledge this is the only prospective study on gastrointestinal cancer risk with information on calcium and albumin as well as other comorbidities. Nevertheless, a direct measure of serum ionized calcium should have been preferable but is seldom used in clinical practice due to high measurement costs but albumin-corrected serum calcium is a valid assessment of ionized calcium on a group level [16]. This database provided complete follow-up for each person as well as linkage to other registers allowing for detailed information on cancer diagnosis, time of death, and emigration. The AMORIS population was selected by analysing blood samples from health check-ups in non-hospitalised individuals. The AMORIS cohort is similar to the general working population of Stockholm in terms of SES and ethnicity. During the study period all-cause mortality was about 14% lower in the AMORIS population than in the general population of Stockholm county when taking age, gender, and calendar year into account [34]. This selection of a healthy cohort does however not affect the internal validity of our study. To account for detection bias, we adjusted all analyses for other comorbidities (CCI). High serum levels of calcium can bias the results because these persons receive more intensive medical care (e.g. increased number of clinical visits due to other diseases and symptoms related to high calcium levels [5] and are thus more likely to be diagnosed with gastrointestinal cancer when it occurs. It is also possible that gastrointestinal cancer events are underestimated due to “early death” from other diseases related with high serum levels of calcium, such as cardiovascular death [7]. However, age-stratified analyses with additional adjustments for the CCI minimized the possible effect of competing risks. A restriction of this study is that limited data on BMI was available, which confines the statistical power of our stratified analyses. There was also no information available on potential confounders such as supplemental use of vitamin D and/or calcium. Furthermore, we were unable to account for the effect of dietary calcium on gastrointestinal carcinogenesis as suggested in biological studies [23]. There was also no information available on tumour grade, stage, or histology making it impossible to assess the association between calcium and tumour severity, but gastrointestinal cancer-specific death can be seen as a proxy for tumour severity.

## Conclusion

Serum calcium was positively associated to risk of oesophageal and colon cancer alone as well as colorectal cancer in women, opposing prior evidence from nutritional studies. This may be caused by different biological mechanisms linking dietary calcium and serum calcium with gastrointestinal carcinogenesis. Our findings also suggest that adjustment or correction based on albumin is important in observing the true association between calcium and gastrointestinal cancer risks. Finally, future studies on the role of calcium metabolism and gastrointestinal cancer development need to assess not only serum calcium but also dietary intake and factors regulating calcium homeostasis, e.g. serum vitamin D and PTH.

## Competing interest

The authors declared that they have no competing interest.

## Authors’ contributions

WW designed the study, analyzed the data, interpreted analysis results, and wrote the paper. KM interpreted analysis results and edited the manuscript. HG, NH, IJ, GW, ML, LH contributed to the analysis tools and database used in this study and edited the manuscript. MVH conceived and designed the study, interpreted analysis results, and edited the manuscript. All authors read and approved the final manuscript.

## Pre-publication history

The pre-publication history for this paper can be accessed here:

http://www.biomedcentral.com/1471-2458/13/663/prepub
